# Low RBM3 protein expression correlates with tumour progression and poor prognosis in malignant melanoma: An analysis of 215 cases from the Malmö Diet and Cancer Study

**DOI:** 10.1186/1479-5876-9-114

**Published:** 2011-07-21

**Authors:** Liv Jonsson, Julia Bergman, Björn Nodin, Jonas Manjer, Fredrik Pontén, Mathias Uhlén, Karin Jirström

**Affiliations:** 1 Department of Clinical Sciences, Pathology, Lund University, Skåne University Hospital, 221 85 Lund, Sweden; 2 Department Clinical Sciences, Surgery, Lund University, Skåne University Hospital, 205 02 Malmö, Sweden; 3 The Malmö Diet and Cancer Study, Lund University, 205 02 Malmö, Sweden; 4 Department of Genetics and Pathology, Rudbeck Laboratory, Uppsala University, 251 87 Uppsala, Sweden; 5 Department of Proteomics, AlbaNova University Center, Royal Institute of Technology, 106 91 Stockholm, Sweden; 6 Science for Life Laboratory, Royal Institute of Technology, 106 91 Stockholm, Sweden

## Abstract

**Background:**

We have previously reported that expression of the RNA- and DNA-binding protein RBM3 is associated with a good prognosis in breast cancer and ovarian cancer. In this study, the prognostic value of immunohistochemical RBM3 expression was assessed in incident cases of malignant melanoma from a prospective population-based cohort study.

**Methods:**

Until Dec 31^st ^2008, 264 incident cases of primary invasive melanoma had been registered in the Malmö Diet and Cancer Study. Histopathological and clinical information was obtained for available cases and tissue microarrays (TMAs) constructed from 226 (85.6%) suitable paraffin-embedded tumours and 31 metastases. RBM3 expression was analysed by immunohistochemistry on the TMAs and a subset of full-face sections. Chi-square and Mann-Whitney U tests were used for comparison of RBM3 expression and relevant clinicopathological characteristics. Kaplan Meier analysis and Cox proportional hazards modelling were used to assess the relationship between RBM3 and recurrence free survival (RFS) and overall survival (OS).

**Results:**

RBM3 could be assessed in 215/226 (95.1%) of primary tumours and all metastases. Longitudinal analysis revealed that 16/31 (51.6%) of metastases lacked RBM3 expression, in contrast to the primary tumours in which RBM3 was absent in 3/215 (1.4%) cases and strongly expressed in 120/215 (55.8%) cases. Strong nuclear RBM3 expression in the primary tumour was significantly associated with favourable clinicopathological parameters; i.e. non-ulcerated tumours, lower depth of invasion, lower Clark level, less advanced clinical stage, low mitotic activity and non-nodular histological type, and a prolonged RFS (RR = 0.50; 95% CI = 0.27-0.91) and OS (RR = 0.36, 95%CI = 0.20-0.64). Multivariate analysis demonstrated that the beneficial prognostic value of RBM3 remained significant for OS (RR = 0.33; 95%CI = 0.18-0.61).

**Conclusions:**

In line with previous in vitro data, we here show that RBM3 is down-regulated in metastatic melanoma and high nuclear RBM3 expression in the primary tumour is an independent marker of a prolonged OS. The potential utility of RBM3 in treatment stratification of patients with melanoma should be pursued in future studies.

## Background

Malignant melanoma is an aggressive form of cancer with a variable clinical course even in patients with thin melanomas and localized disease [1-4]. Despite increasing insights into melanoma biology and the discovery of gene- and protein-signatures that supplement established prognostic clinicopathological parameters [5-7], no biomarkers have yet been incorporated into clinical protocols.

The RNA-binding motif protein 3, RBM3, was initially identified in a human fetal brain tissue cDNA library [8]. The RBM3 gene maps to Xp11.23 and encodes two alternatively spliced RNA transcripts. RBM3 transcripts have been found in various human tissues [8] and in vitro, RBM3 is one of the earliest proteins synthesized in response to cold shock [9]. RBM3 contains one RNA-recognition motif (RRM) and is able to bind to both DNA and RNA, whereby a glycine rich region adjacent to the RNA binding motif is thought to enhance the protein-RNA or protein-DNA interaction [8,10].

Based on an initial discovery in the Human Protein Atlas (HPA) http://www.proteinatlas.org[11-13], we have recently demonstrated that tumour-specific expression of RBM3, in particular its nuclear localization, is associated with a significantly improved survival in breast cancer [14] and ovarian cancer [15], and that RBM3 confers cisplatin sensitivity in ovarian cancer cells [15]. Apart from these studies, we are not aware of any other publications related to the prognostic or treatment predictive impact of the tumour-specific expression of RBM3 in human cancer, and the biological processes underlying these observations have not yet been unraveled. It is evident that RBM3 is up-regulated in various types of human malignancies [14,16,17] and *in vitro *studies in a wide range of different model systems have demonstrated that RBM3 is involved in multiple processes central to cancer biology, like proliferation [15-17], apoptosis [18,19] and angiogenesis [16].

The prognostic value of RBM3 expression has, to our knowledge, not yet been investigated in malignant melanoma. However, down-regulation of RBM3 at the gene expression level has been demonstrated in an *in vitro *model of melanoma progression [20].

In the present study, we investigated the prognostic impact of immunohistochemical (IHC) RBM3 expression in 215 incident malignant melanomas in the prospective, population-based cohort Malmö Diet and Cancer Study (MDCS) [21]. For this purpose, tissue microarrays (TMAs) were constructed from suitable tumours (n = 226) and a subset of metastases (n = 31). It is demonstrated that strong nuclear expression of RBM3 correlates with favourable clinicopathological parameters and independently predicts a significantly prolonged overall survival. In addition, a markedly reduced expression of RBM3 was observed in metastases compared to primary tumours, which is quite in line with previous *in vitro *data [20].

## Methods

### The Malmö Diet and Cancer Study

The Malmö Diet and Cancer Study (MDCS) is a population-based prospective cohort study with the main aim to examine whether a Western diet rich in fat and low in fruit and vegetables increases the risk of certain forms of cancer. Between 1991-1996, a total number of 28 098 individuals; 11 063 (39,4%) men and 17 035 (60,6%) women between 44-74 years where enrolled (from a background population of 74 138). All participants completed the baseline examination, which included a questionnaire, measures of anthropometric/body compositions and a dietary assessment. The questionnaire covered questions on physical activity, use of tobacco and alcohol, heredity, socio-economic factors, education, occupation, previous and current disease and current medication. In addition, blood samples were collected and stored in -80°C. Follow up is done annually by record-linkage to national registries for cancer and cause of death [22].

Ethical permissions for the MDCS (Ref. 51/90) and the present study (Ref. 530/2008) were obtained from the Ethical Committee at Lund University.

### Incident malignant melanomas until Dec 31^st ^2008

Until end of follow-up 31 December 2008, 264 incident invasive malignant melanomas had been registered in the study population. Cases were identified from the Swedish Cancer Registry up until 31 Dec 2007, and from The Southern Swedish Regional Tumour Registry for the period of 1 Jan-31 Dec 2008. All tumours with available slides and/or paraffin blocks were histopathologically re-evaluated on haematoxylin and eosin stained slides whereby information on lymphocytic infiltration (none, mild, moderate or high), ulceration (absent or present), mitotic count and vascular invasion was obtained. Data on location, Clark level and Breslow depth of invasion was obtained from the clinical- and/or pathology records.

Information on recurrence (local, regional or distant) was obtained in 2010 from patient records and pathology reports. Information on vital status and cause of death was obtained from the Swedish Cause of Death Registry up until 31 Dec 2009.

### Tissue microarray construction

Paraffin-embedded tumour specimens were collected from the archives of the pathology departments in the region of Skåne in Southern Sweden. Tumours with an insufficient amount of material were excluded. Areas representative of cancer were then marked on haematoxylin & eosin stained slides and TMAs constructed as previously described [23]. In brief, three 0,6 mm cores were taken from each tumour and mounted in a new recipient block using semi-automated arraying device (TMArrayer, Pathology Devices, Westminster, MD, USA). In addition, metastases (representing both regional and distant metastases in various organs) were sampled from 31 cases. Thin melanomas (< 1 mm) were subjected to TMA construction if the diameter was > 1 cm. To check for heterogeneity, IHC staining was also performed on additional full-face sections from 25 cases.

### Immunohistochemistry and evaluation of RBM3 staining

For immunohistochemical analysis, 4 μm TMA-sections were automatically pre-treated using the PT-link system (DAKO, Glostrup, Denmark) and then stained in a Autostainer Plus (DAKO, Glostrup, Denmark) with the mouse monoclonal anti-RBM3 antibody (AAb030038; Atlas Antibodies AB, Stockholm, Sweden, diluted 1:5000). The specificity of the antibody has been validated previously [15].

As RBM3, when present, was expressed in > 75% of the cells, predominantly in the nuclei and in varying intensities, only the intensity of the staining was accounted for and denoted a score from 0 (negative), 1 (mild), 2 (moderate) and 3 (strong). The staining was evaluated by three independent observers (LJ, JB, and BN) who were blinded to clinical and outcome data. Scoring differences were discussed in order to reach consensus.

### Statistical analysis

Chi-square and Mann-Whitney U tests were used for comparison of RBM3 expression and relevant clinicopathological characteristics. Recurrence was defined as local, regional or distant recurrence or death from malignant melanoma and risk of recurrent disease was referred to as recurrence free survival (RFS). Follow-up started at date of diagnosis and ended at recurrent disease, death, lost to follow-up (emigration) or last date of follow- up with regard to recurrent disease. No recurrences were recorded following the last date of follow-up regarding death, i.e. 31 Dec 2009. Overall survival (OS) was assessed by calculating the risk of death from all causes, overall mortality. Follow-up started at date of diagnosis and ended at death, emigration or 31 Dec 2009, whichever came first.

Kaplan-Meier analysis and log rank test were used to illustrate differences in RFS and OS. Cox regression proportional hazards models were used to estimate the impact of the investigated parameters on RFS and OS in both uni- and multivariate analysis. Some subjects had no information on one or several markers and missing values were coded as a separate category for categorical variables and as the mean of all observations for continuous variables. Missing values for categorical variables co-varied and the multivariate model did not converge due to many constant values. In order to avoid this, the multivariate analysis only included patients with information on RBM3. In addition, the patient with missing information on lymphocytic infiltration had to be excluded. Co-variates were entered into the multivariate analysis using backward selection were a p-value of 0.05 decided entry and a p-value of 0.20 was used for removal. RBM3 was included in all models irrespective of the backward selection procedure.

All tests were two sided. A p-value of 0.05 was considered significant. All statistical analyses were performed using SPSS version 17 (SPSS Inc, Chicago, IL).

### REMARK criteria

A description of the fulfilment of REMARK [24] criteria for biomarker studies is provided in Additional file 1, Table S1.

## Results

### Distribution of clinicopathological parameters in the cohort

The distribution of patient- and tumour characteristics in the full cohort is shown in Table 1. In line with the relatively high median age of cases (69 years, range 44-85), the frequency of lentigo maligna melanomas was slightly higher (11.7%) than the average expected around 7% in Sweden [2]. The proportion of thinner melanomas (< = 1 mm, Stages 1A-B) was also higher than expected (86.5% compared to ~55.1%) as well as the proportion of non-ulcerated tumours (14.1% compared to ~24.1%)[2].

**Table 1:** Patient and tumour characteristics in the full cohort (n = 264)

Age	
Mean	67.29
Median	69.00
Range	46-84
**Sex**	
Female	135 (51.1%)
Male	129 (48.9%)
**Location**	
Head and neck	17 (14.9%)
Extremities	111 (44.8%)
Dorsal thorax	65 (26.2%)
Frontal thorax	35 (14.1%)
*Unknown*	16
**Clark level**	
II	93 (37.7%)
III	103 (41.7%)
IV	44 (17.8%)
V	7 (2.8%)
*Unknown*	17
**Breslow (mm)**	
Mean	1.57
Median	0.71
Range	0.08-40.00
**Breslow AJCC categories**	
< = 1	158 (59.8%)
1-2	36 (13.6%)
2-4	40 (15.2%)
> 4	14 (5.3%)
*Unknown*	16 (6.1%)
**Clinical Stage**	
1A	154 (74.4%)
1B	25 (12.1%)
2A	17 (8.2%)
2B	7 (3.4%)
3A	2 (1.0%)
4	2 (1.0%)
*Unknown*	57
**Histological type**	
SSM	160 (64.5%)
NMM	53 (21.48%)
LMM	29 (11.7%)
Other	6 (2.4%)
*Unknown*	16
**Ulceration**	
Absent	214 (85.9%)
Present	35 (14.%)
*Unknown*	16
**Mitotic count**	
< 1/mm^2^	131(51.8)
> = 1/mm^2^	122(46.2)
**Lymphocytic infiltration**	
None-mild	72 (29.0%)
Moderate-high	176 (66.7%)
Unknown	16
**Recurrence**	
No	217 (82.2%)
Yes	47 (17.8%)
**Follow-up (years)**	
Mean	7.25
Median	6.88
Range	0.64-17.05
**Vital status**	
Dead	55 (20,8%)
Alive	219 (79,2%)
Dead from malignant melanoma	28 (10.6%)

### Immunohistochemical expression of RBM3 in primary tumours and metastases

**Figure 1:**
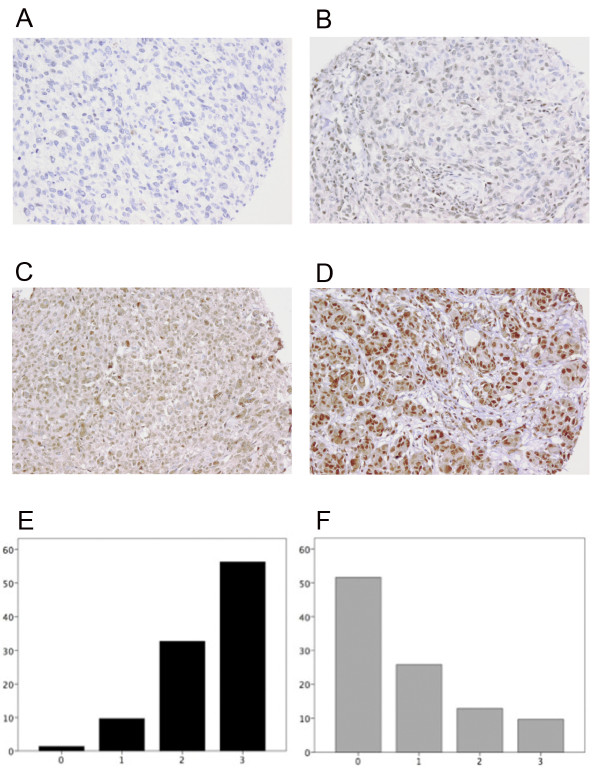
**RBM3 expression in primary melanomas and metastases**. Examples of malignant melanomas with (A) negative, (B) weak, (C) intermediate and (D) strong immunohistochemical RBM3 staining. RBM3 expression was strong in the majority of (E) primary tumours compared to (F) metastases.

Of the 226 cases in the TMA cohort it was possible to evaluate the expression of RBM3 protein in 215 cases (95,1%). There was no obvious heterogeneity in the staining pattern between the tissue cores. There was an excellent concordance between RBM3 scores assessed on full-face sections and TMAs (kappa-value 0.85). Examples of immunohistochemical staining are shown in Figure 1A-D and the staining distribution in primary tumours vs metastases in Figure 1E-F. Interestingly, and in line with previous in vitro data [20], RBM3 expression was strong in the majority of primary tumours, but weak or absent in the metastases (Figure 1E-F). Notably, similar associations were seen when comparing primary tumours and metastases in the 31 cases, for which both locations had been sampled (data not shown).

### Association between RBM3 expression and clinicopathological parameters

As shown in Table 2, there was a strong association between low RBM3 expression and depth of invasion, Clark level, clinical stage, mitotic count, nodular vs non-nodular type and ulceration. However, localization, age, lymphocytic infiltration and melanoma type were not associated with RBM3 expression. In some cases with strong RBM3 expression, cytoplasmic staining was present in various intensities, but this did not add any prognostic value (data not shown).

**Table 2:** Association between RBM3 expression and clinicopathological parameters

			
	**RBM3 staining intensity**		
	0-2	3	
n(%)	95 (44.2)	120 (55.8)	p-value

**Age**			
Mean	68.00	67.15	0.673
(range)	(47-83)	(46-81)	
			
**Gender**			
Female	45(47.4)	63(52.5)	0.457
Male	50(52.6)	57(47.5)	
			
**Clark level**			
II	21(22.3)	50(42.4)	0.005**
IIII	48(51.1)	46(39.0)	
IV-V	25(26.6)	22(18.6)	
*missing*	*1*	*2*	
			
**Breslow(mm)**			
Mean	2.47	1.12	0.001**
(range)	(0.08-40.00)	(0.11-7.00)	
			
**Ulceration**			
No	71(74.7)	109(91.6)	0.001**
Yes	24(25.3)	10(8.4)	
*missing*	*0*	*1*	
			
**Lymphocytic**			
**infiltrate**			
0-1	26(27.4)	34(28.6)	0.846
2-3	69(72.6)	85(71.4)	
*missing*	*0*	*1*	
			
**Clinical stage**			
I	54(76.1)	95(91.3)	0.005*
II-IV	17(23.9)	9(8.7)	
*missing*	*24*	*16*	
			
**Vascular invasion**			
No	87(91.6)	114(95.0)	0.314
Yes	8(8.4)	6(5.0)	
			
**Localization**			
Head and neck	15(16.0)	16(14.0)	0.352
Extremities	40(42.6)	59(51.8)	
Frontal thorax	10(10.6)	15(13.2)	
Dorsal thorax	29(30.9)	24(21.1)	
*missing*	*1*	*6*	
			
**Type**			
SSM, LMM, Other	65(58.9)	97(68.9)	0.027*
NMM	30(31.6)	22(18.5)	
*missing*	*0*	*1*	
			
**Mitotic count**			
< 1/mm^2^	34(35.8)	71(59.2)	0.001**
> = 1/mm^2^	61(64.2)	49(40.8)	
			

*Significant at the 0.05 level

** Significant at the 0.01 level

§ Mann Whitney U test for comparison of means

SSM = Superficial spreading melanoma

NMM = Nodular malignant melanoma

LMM = Lentigo malignant melanoma

### Impact of high RBM3 expression on recurrence free survival and overall survival

Having demonstrated that RBM3 is associated with less advanced disease and favourable clinicopathological parameters, the relationship between RBM3 expression and disease outcome was examined. For survival analysis, data were dichotomized into strong vs negative-moderate intensity for RBM3.

**Figure 2:**
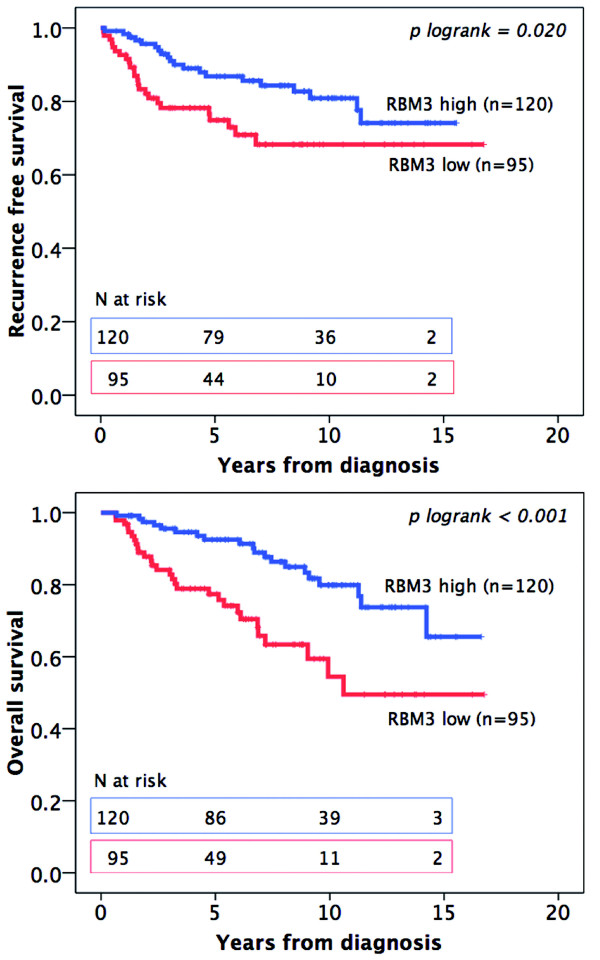
**Prognostic value of RBM3 expression in primary melanoma**. Tumours with high (strong intensity) RBM3 expression had a significantly improved (A) recurrence free survival and (B) overall survival compared to tumours with low RBM3 expression (negative to moderate intensity).

Kaplan Meier analysis of the evaluated cohort (n = 215) demonstrated that high expression of RBM3 was associated with a significantly prolonged RFS (p = 0.020) and OS (p < 0.001) (Figure 2). In Cox multivariate analysis, high RBM3 expression remained an independent prognostic parameter for OS but not RFS (Table 3).

**Table 3:** Relative risks of recurrence and death according to clinicopathological parameters and RBM3 expression

		Relative risk of recurrence			Relative risk of death	
				
		Univariate	Multivariate		Univariate	Multivariate
	*n(events)*	RR(95%CI)	RR(95%CI)	*n(events)*	RR(95%CI)	RR(95%CI)
						
**Age**						
Continuous	*255(47)*	1.01(0.97-1.05)		*255(53)*	1.09(1.04-1.13)	1.07(1.02-1.12)
						
**Gender**						
Female	*132(21)*	1.00		*132(18)*	1.00	1.00
Male	*123(26)*	1.51(0.85-2.69)			2.60(1.47-4.61)	2.37(1.22-4.57)
						
**Clark level**						
II	*93(5)*	1.00	1.00	*93(13)*	1.00	
III	*103(21)*	4.39(1.65-11.65)	1.93(0.51-7.26)	*103(21)*	1.70(0.85-3.40)	
IV-V	*51(21)*	9.99(3.76-26.55)	1.02(0.24-4.34)	*51(18)*	3.04(1.49-6.22)	
						
**Breslow**						
Continuous	*248(47)*	1.07(1.03-1.11)		*248(53)*	1.07(1.03-1.12)	
						
**Subtype**						
SSM, LMM, Other	*195(22)*	1.00		*195(30)*	1.00	1.00
Nodular	*53(24)*	5.63(3.15-10.08)		*53(22)*	3.86(2.21-6.74)	2.32(1.20-4.94)
						
**Ulceration**						
No	*213(30)*	1.00		*214(35)*	1.00	1.00
Yes	*35(16)*	5.80(3.12-10.77)		*35(17)*	6.29(3.46-11.45)	3.52(1.63-7.61)
						
**Lymphocytic**						
**infiltrate**						
0-1	*72(20)*	1.00		*72(22)*	1.00	1.00
2-3	*176(76)*	0.43(0.24-0.78)		*176(30)*	0.46(0.26-0.79)	0.55(0.30-1.00)
						
**Clinical stage**						
I	*179(12)*	1.00	1.00	*179(22)*	1.00	
II-IV	*28(13)*	15.02(6.39-35.30)	7.36(2.47-21.47)	*28(11)*	6.48(3.05-13.73)	
						
**Mitotic count**						
< 1/mm^2^	*131(7)*	1.00	1.00	*131(17)*	1.00	
> = 1/mm^2^	*122(40)*	7.99(3.56-17.80)	2.86(0.96-8.47)	*122(36)*	1.26(1.19-1.34)	
						
**Vascular invasion**						
No	*232(35)*	1.00	1.00	*232(42)*	1.00	1.00
Yes	*14(11)*	9.25(4.67-18.35)	3.40(1.60-7.20)	*14(9)*	4.88(2.37-10.08)	3.81(1.62-8.97)
						
**RBM3 intensity**						
0-2	*95(24)*	1.00	1.00	*95(29)*	1.00	1.00
3	*120(20)*	0.50 (0.27-0.91)	0.87(0.46-1.66)	*120(20)*	0.36(0.20-0.64)	0.33(0.18-0.61)
						

The number of cases in the multivariate analysis is equal to the number of cases evaluated for RBM3 expression (n = 215).

In thin melanomas (< = 1 mm; n = 129) there was no significant association between RBM3 expression and RFS (data not shown) and a trend, however non-significant, towards a prolonged OS for tumours with high RBM3 expression (RR = 0.48; 95%CI = 0.18-1.24). In melanomas > 1 mm (n = 84), RBM3 was not associated with RFS (data not shown) but with a significantly improved OS (RR = 0.40; 95% CI = 0.19-0.85), which remained significant in multivariate analysis (RR = 0.29; 95% CI = 0.11-0.77). Notably, tumour thickness measured as a continuous variable did not remain significant in multivariate analysis. However, this was not altered when AJCC categories (< 1 mm, 1-2 mm, 2-4 mm and > 4 mm) were used instead or when clinical stage was excluded from the analysis (data not shown).

Information on tumour diameter was only available for 162 (61%) of the patients and therefore not included in the analyses. There was an inverse association between tumour diameter and RBM3 expression (p = 0.030) but not to depth of invasion (data not shown). There was no association between tumour diameter and survival (data not shown)

## Discussion

This study provides a first description of the patient and tumour characteristics of incident cases of malignant melanoma in the prospective, population-based cohort Malmö Diet and Cancer Study, diagnosed until Dec 31^st^, 2008. In addition, it is demonstrated that the investigative biomarker RBM3 is down-regulated in metastatic deposits, associated with favourable histopathological parameters in primary melanomas and an independent predictor of a prolonged overall survival. In a translational context, these findings are quite in line with a previous study, where RBM3 was demonstrated to be one of five down-regulated genes in an *in vitro *model of melanoma progression [20]. Moreover, as RBM3 has been demonstrated to be a good prognostic biomarker in several other cancer forms, e.g. breast cancer [14] and ovarian cancer [15], its clinical utility in stratification of melanoma patients should be validated in future studies.

According to current clinical guidelines in Sweden, sentinel node biopsy is performed in melanomas > 1 mm, but as an increase in thin melanomas (< = 1 mm) seems to make up for most of the increasing incidence of malignant melanomas [25], there is an unmet need for prognostic biomarkers in this category [26]. In this study, RBM3 was not significantly associated with prognosis in thin (< = 1 mm) melanomas but was an independent favourable prognostic factor for OS in melanomas > 1 mm. The reason for this remains unclear and further studies in larger patient cohorts are needed to determine the prognostic value of RBM3 in thin melanomas. However, the observation that RBM3 remained an independent factor for overall survival in the cohort as a whole, which represented tumours of less advanced clinical stages than in the average population [2], indicates its potential utility as a biomarker for prognostic stratification of patients with early-stage melanoma.

In the light of the above, a methodological aspect that needs further attention is the bias related to the use of the TMA technique in malignant melanoma biomarker studies, e.g. the technical difficulty in sampling small tumours. In this study, we attempted to sample melanomas < 0.5 mm if the diameter was > 10 mm, and in several cases, sampling was successful. The mean Breslow depth of invasion in the TMA cohort was only slightly higher than in the full cohort (1.66 mm compared to 1.57 mm). In addition, as determined by comparison with full-face sections for a subset of the tumours, RBM3 did not seem to display a heterogeneous expression pattern.

In this study we used a monoclonal antibody against RBM3, which was also used in our previous study on ovarian cancer [15]. In the first paper, describing the prognostic value of RBM3 in breast cancer, we used a polyclonal antibody generated within the HPA project [14]. Both antibodies have been extensively validated using siRNA techniques in breast cancer cell lines [14] and ovarian cancer cell lines [15] and similar results have been obtained regarding the staining distribution in various normal and cancerous tissues (data not shown). Although being a semi-quantitative method, IHC has several advantages since it allows for assessment of protein expression in different sub-cellular compartments, which might have important prognostic implications. In the case of RBM3, previous findings indicate that its nuclear rather than cytoplasmic localization is the most relevant parameter for prognostication [14,15], which is also demonstrated here for melanoma.

As the MDCS is a population-based cohort study, a potential selection bias compared to the general population must be taken into consideration [22]. Since all participants were > 40 years at study entry, the mean age among melanoma cases was higher than in the average population. Notably, since older melanoma patients often present with more advanced disease [27], the relatively low proportion of cases with advanced disease reported here is somewhat unexpected. This could in part be explained by the fact that data necessary for staging could not be obtained for all cases. Nevertheless, clinical stage, as well as the prognostic impact of other established clinicopathological characteristics fell out as expected, which validates the cohort as a platform for future studies of lifestyle and tumour biology in relation to melanoma risk and prognosis.

Given the previously demonstrated association between RBM3 and cisplatin sensitivity in ovarian cancer cell lines [15], the potential value of RBM3 as a predictor of response to platinum-based chemotherapy in patients with metastatic malignant melanoma could be of interest to investigate in future studies. However, in contrast to the situation in ovarian cancer, where RBM3 showed a consistent expression pattern in primary tumours and omental deposits [15], the data presented here, and previous in vitro data [20], show that RBM3 is down-regulated in the majority of metastatic melanomas. Hence, in the predictive setting in melanoma patients, thorough sampling and immunohistochemical analysis of metastatic deposits would be required in order to identify a comparatively small number of patients with RBM3 positive metastases.

## Conclusions

We have demonstrated that the RNA- and DNA-binding protein RBM3 is an independent biomarker of a prolonged OS in patients with primary malignant melanoma and that RBM3 expression is lost during progression of the disease. The potential utility of RBM3 in risk stratification of patients with melanoma should be pursued in future studies.

## Competing interests

The authors declare that they have no competing interests.

## Authors' contributions

LJ and JB participated in the data collection, performed the statistical analysis and drafted the manuscript. BN assisted with the data collection, constructed the tissue microarrays and helped draft the manuscript. JM, FP and MU participated in the design of the study and helped draft the manuscript. KJ conceived of the study, participated in its design and coordination and helped to draft the manuscript. All authors read and approved the final manuscript.

## Supplementary Material

Additional file 1 **Supplementary Table 1**. Fulfilment of REMARK criteria [24]. Click here for file
