# Supplementary material for: Low RBM3 protein expression correlates with tumour progression and poor prognosis in malignant melanoma: An analysis of 215 cases from the Malmö Diet and Cancer Study
Source: J Transl Med. 2011 Jul 21;9:114. doi: 10.1186/1479-5876-9-114 (PMC3156749; doi:10.1186/1479-5876-9-114)
Supplement: Additional file 1 — Supplementary Table 1. Fulfilment of REMARK criteria [24]. [file 1479-5876-9-114-S1.DOCX]

| **Reporting recommendations for REMARK** | **How criteria are fulfilled** |
| --- | --- |
| **Introduction** |  |
| 1. State the marker examined, the study objectives, and any pre-specified hypotheses | The hypothesis and study objectives are stated on pp 5-6 |
| **Materials and Methods** |  |
| *Patients* |  |
| 1. Describe the characteristics of study patients, including their source and inclusion and exclusion criteria. | Table 1 describes the characteristics of the study patients. This is a population based, prospective cohort study. Details of the study population are described on pp 7-8. |
| 2.Describe treatments received and how chosen | Not applicable |
| *Specimen Characteristics* |  |
| 1. Describe type of biological material used and methods of preservation and storage | The biomarker study is performed on archival paraffin-embedded tumour material, from which suitable specimens were assembled in tissue microarrays all stored and handled in room-temperature. Described on p 8 |
| 2. Specify the assay used and provide (or reference) a detailed protocol, including specific reagents or kits used, quality control procedures, reproducibility assessments, quantitation methods, and scoring and reporting protocols. Specify whether and how assays were performed blinded to the study endpoint. | Details of assays and protocols used are given on pp 8-9.  Assays were performed blinded to the study endpoint |
| *Study Design* |  |
| 1. State the method of case, selection including whether prospective or retrospective and whether stratification or matching was used. Specify the time period from which cases were taken, the end of the follow-up period, and the median follow-up time. | Details of case selection and the time period are given on pp 7-8. |
| 2. Precisely define all clinical endpoints examined. | Details of endpoints are given on pp 7-9. |
| 3. List of all candidate variables initially examined for inclusion in models. | Not applicable as there was only one candidate variable in this study. |
| 4. Give rationale for sample size; if the study was designed to detect a specified effect size, give the target power and effect size. | Not applicable since this study involved incident melanoma cases in a prospective, population-based cohort study. |
| *Statistical analysis methods* |  |
| 5. Specify all statistical methods, including details of any variable selection procedures and other model-building issues, how model assumptions were verified, and how missing data were handled. | Specified on p9 |
| 6. Clarify how marker values were handled in the analysis. | Clarified on pp7-11 |
| **Results** |  |
| *Data* |  |
| 1. Describe the flow of patients through the study, including the number of patients included in each stage of the analysis and reasons for drop out. Specifically, report the number of patients and the number of events. | The flow of patients is described on pp7-8. This was not a staged analysis. |
| 2. Report distributions of basic demographic characteristics, standard prognostics variables | Table 1 shows patient and tumour characteristics in the full cohort and Table 2 in the tissue microarray cohort. Standard prognostic indicators are also included in Table 3. |
